# Interactions of Respiratory Viruses and the Nasal Microbiota during the First Year of Life in Healthy Infants

**DOI:** 10.1128/mSphere.00312-16

**Published:** 2016-11-23

**Authors:** Insa Korten, Moana Mika, Shkipe Klenja, Elisabeth Kieninger, Ines Mack, Maria Teresa Barbani, Meri Gorgievski, Urs Frey, Markus Hilty, Philipp Latzin

**Affiliations:** a Division of Respiratory Medicine, Department of Pediatrics, Inselspital, Bern University Hospital, University of Bern, Bern, Switzerland; b Graduate School for Cellular and Biomedical Sciences, University of Bern, Bern, Switzerland; c University of Basel, Children’s Hospital (UKBB), Basel, Switzerland; d Institute for Infectious Diseases, University of Bern, Bern, Switzerland; e Department of Infectious Diseases, University Hospital Bern, Bern, Switzerland; University of Kentucky College of Medicine

**Keywords:** bacteriology, human rhinovirus, microbiota, pediatric infectious disease, respiratory viruses

## Abstract

Traditional culture techniques have shown that increased bacterial colonization is associated with viral colonization; however, the influence of viral colonization on the whole microbiota composition is less clear. We thus aimed to understand the interaction of viral infections and the nasal microbiota in early life to appraise their roles in disease development. Thirty-two healthy, unselected infants were included in this prospective longitudinal cohort study within the first year of life. Biweekly nasal swabs (*n* = 559) were taken, and the microbiota was analyzed by 16S rRNA pyrosequencing, and 10 different viruses and 2 atypical bacteria were characterized by real-time PCR (combination of seven duplex samples). In contrast to asymptomatic human rhinovirus (HRV) colonization, symptomatic HRV infections were associated with lower alpha diversity (Shannon diversity index [SDI]), higher bacterial density (PCR concentration), and a difference in beta diversities (Jaccard and Bray-Curtis index) of the microbiota. In addition, infants with more frequent HRV infections had a lower SDI at the end of the study period. Overall, changes in the microbiota associated with symptomatic HRV infections were characterized by a loss of microbial diversity. The interaction between HRV infections and the nasal microbiota in early life might be of importance for later disease development and indicate a potential approach for future interventions.

**IMPORTANCE** Respiratory viral infections are very frequent in infancy and of importance in acute and chronic disease development. Infections with human rhinovirus (HRV) are, e.g., associated with the later development of asthma. We found that only symptomatic HRV infections were associated with acute changes in the nasal microbiota, mainly characterized by a loss of microbial diversity. Infants with more frequent symptomatic HRV infections had a lower bacterial diversity at the end of the first year of life. Whether the interaction between viruses and the microbiota is one pathway contributing to asthma development will be assessed in the follow-ups of these children. Independent of that, measurements of microbial diversity might represent a potential marker for risk of later lung disease or monitoring of early life interventions.

## Abstract (precis)

Respiratory viral infections are very frequent in infancy and of importance in acute and chronic disease development. Infections with human rhinovirus (HRV) are, e.g., associated with the later development of asthma. We found that only symptomatic HRV infections were associated with acute changes in the nasal microbiota, mainly characterized by a loss of microbial diversity. Infants with more frequent symptomatic HRV infections had a lower bacterial diversity at the end of the first year of life. Whether the interaction between viruses and the microbiota is one pathway contributing to asthma development will be assessed in the follow-ups of these children. Independent of that, measurements of microbial diversity might represent a potential marker for risk of later lung disease or monitoring of early life interventions.

## INTRODUCTION

Recent evidence implies that the respiratory microbiota in early life plays an important role in shaping the immune system as well as in chronic respiratory diseases (1–6). A disordered microbiota has been reported, for example, in asthma and cystic fibrosis (CF) (7, 8); the role of the microbiota in disease development is, however, less clear (9). A recent study has shown that in healthy infants, age and season play an important role (10), but little is known about associations with or factors influencing the respiratory microbiota in early life.

An important and established factor for the later onset of asthma is the occurrence of respiratory viral infections, above all human rhinovirus (HRV) and respiratory syncytial virus (RSV) (11). Furthermore, respiratory viral infections play an important role in morbidity and early immune development during the first year of life in otherwise healthy infants (12–16). Despite recent data on the association between viral infections and changes in the microbiota in adults, data for young children are scarce (17–22). Due to several risk factors triggering both viral and bacterial infections, it is difficult to draw causal associations between those early infections and later disease development. However, based on the long-term importance of effects early in life, the exact interaction of viral colonization and microbiota in infancy seems to be of great interest.

The aim of our study was thus to assess a possible association between viral colonization and the nasal microbiota in unselected, healthy infants within the first year of life. We hypothesized that the presence of a virus will change the composition of the microbiota.

## RESULTS

### Study population.

**FIG 1:**
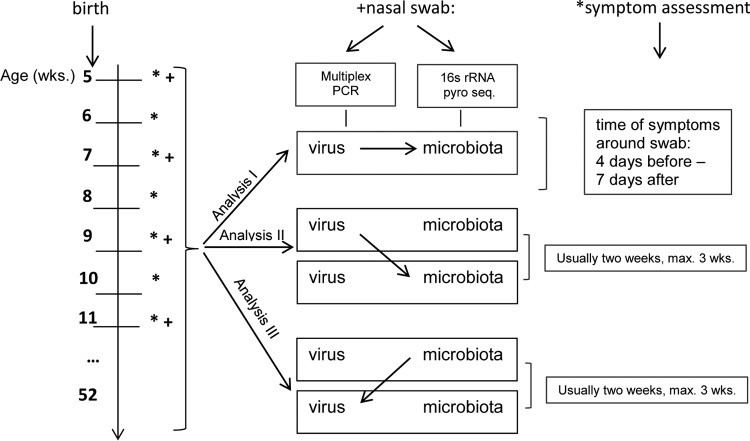
Study design. *, weekly telephone calls with standardized interviews regarding symptoms of lower and upper respiratory infections, wheeze, and/or cough; +, biweekly analysis of nasal swab samples, including analysis of the respiratory microbiota by PCR amplification of bacterial 16S rRNA and virological examination with multiplex PCR. 16S rRNA pyro seq., bacterial 16S rRNA pyrosequencing. Detailed explanation of analysis of viral colonization and the microbiota can be found in the Results section. Analysis I examined association of viral colonization and the microbiota in the same sample. Microbiota is used as the outcome parameter. Analysis II examined association of viral colonization and the microbiota within 3 weeks after viral colonization. Microbiota is used as the outcome parameter, and only samples free of virus after viral colonization are included for evaluation. Analysis III examined association of viral colonization and the microbiota within 3 weeks before viral colonization. Virus is used as the outcome parameter, and only samples free of virus before viral colonization are included for evaluation.

Thirty-two healthy, unselected term-born infants completed the study period of 1 year, with a weekly telephone interview to assess respiratory health and biweekly nasal swab sampling by parents. After exclusion of low-quality results for either virus or microbiota, a total of 559 nasal swabs were evaluated both for bacterial and virological analyses (see Fig. S1 in the supplemental material). This corresponds to a mean of 17.5 swabs (range, 11 to 24; standard deviation [SD], 3.3) per infant. For demographics and other risk factors, see Fig. 1 and Table 1 and see Table S1 in the supplemental material.

10.1128/mSphere.00312-16.1 Figure S1  Flowchart of study infants and nasal swab processing for viral and microbiota analysis. Download Figure S1, PDF file, 0.1 MB. Copyright © 2016 Korten et al. 2016 Korten et al. This content is distributed under the terms of the Creative Commons Attribution 4.0 International license.

10.1128/mSphere.00312-16.5 Table S1  Risk factors for HRV colonization. Download Table S1, PDF file, 0.1 MB. Copyright © 2016 Korten et al. 2016 Korten et al. This content is distributed under the terms of the Creative Commons Attribution 4.0 International license.

**TABLE 1:** Characteristics of the study population^a^

Characteristic	Result
Anthropometrics	
Sex (female), no. (%)	13 (41)
Gestational age at birth (wk), mean (SD)	39.8 (1.2)
Length at birth (cm), mean (SD)	49.7 (2.1)
Birth wt (kg), mean (SD)	3.4 (0.5)
Season of birth, no. (%)^b^	
Winter	8 (25)
Spring	7 (22)
Summer	9 (28)
Fall	8 (25)
Measurements	
No. of wk with nasal swabs, mean (range)	17.5 (11–24)

^*a*^*n* = 32 infants.

b Season of birth is categorized with the calendar definition of season: winter, 22 December to 20 March; spring, 21 March to 20 June; summer, 21 June to 22 September; fall, 23 September to 21 December.

### Description of microbiota and virus detection.

The composition of the microbiota in the first year of life was described in detail before (10). Briefly, bacterial density increased and diversity decreased within the first year of life. If investigating the bacterial families, the relative abundance of *Staphylococcaceae* and *Corynebacteriaceae* was highest in the first 3 months of life. A virus was detected in 241 samples (43%); of those, 123 (51%) were accompanied by respiratory symptoms and 11 (5%) samples were taken during periods with more severe respiratory symptoms (lower respiratory tract infection [LRTI]). The most frequent virus was HRV (147 samples [26%]); all of the other viruses analyzed were detected in 39 (7%) samples or less; RSV was found only in 11 samples (2%) (for complete viral colonization, see Table 2). We thus grouped all other viruses as “viruses other than HRV.” After excluding the samples cocolonized with HRV, in 94 (17%) samples, “viruses other than HRV” were detected. Of those, 38 (40%) were accompanied by respiratory symptoms, and 56 (60%) were not. A total of 120 samples (21%) showed HRV colonization without cocolonization with other viruses. Of those, 69 (57.5%) were accompanied by respiratory symptoms, while 51 (42.5%) were not. A total of 318 samples (57%) remained free of any viral colonization; of those, 77 samples (24%) did show respiratory symptoms, and 6 (2%) were taken during LRTI.

**TABLE 2:** Number and percentage of samples tested positive for the different viruses and atypical bacteria

Sample type^a^	Samples with virus		Positive samples accompanied by symptoms	
	*n*	%	*n*	%
Any virus	241	42	123	22
>1 virus	36	6	22	4
HRV/EV	147	26	69	12
HCoV	39	7	7	1
ADV	31	5	6	1
PIV	23	4	7	1
hBoV	12	2	3	<1
RSV	11	2	3	<1
hPEV	8	1	2	<1
hMPV	5	1	2	<1
*M. pneumoniae*	4	<1	1	<0.5
Influenza A virus	1	<0.5	1	<0.5
Influenza B virus	1	<0.5	0	0
*C. pneumoniae*	0	0	0	0

a Total number of nasal swabs, *n* = 559. Coinfections are not considered in samples when symptoms were present. ADV, adenovirus; hBoV, human Bocavirus; HCoV, human coronavirus; hMPV, human metapneumovirus; hPEV, human parechovirus; HRV/EV, rhinovirus/enterovirus; PIV, parainfluenzavirus.

### Changes in the microbiota during viral colonization.

Changes in the microbiota during viral colonization are depicted in analysis I in Fig. 1. HRV colonization was associated with a higher bacterial density and a lower alpha diversity: mean PCR concentration (PCR_concn_), 44 (standard deviation [SD], 35) ng/µl versus 29 [SD, 29] ng/µl; Shannon diversity index (SDI), 0.9 (SD, 0.7) versus 1.3 (SD, 0.9) (Table 3).

**TABLE 3:** Unadjusted and adjusted analysis of the association of HRV colonization with the microbiota in the same sample^a^

Outcome	Unadjusted model			1st adjusted model^b^			2nd adjusted model^c^		
	IRR/Coeff	95% CI	*P*	IRR/Coeff	95% CI	*P*	IRR/Coeff	95% CI	*P*
PCR_concn_									
HRV, no symptoms	1.12	0.88 to 1.41	0.35	1.17	0.92 to 1.48	0.2	1.14	0.90 to 1.44	0.27
HRV, symptoms	1.44	1.19 to 1.76	0	1.38	1.13 to 1.68	0	1.3	1.06 to 1.58	0.01
SDI									
HRV, no symptoms	0.05	−0.06 to 0.17	0.371	0.02	−0.09 to 0.14	0.689	0.03	−0.09 to 0.14	0.677
HRV, symptoms	−0.18	−0.28 to −0.07	0.001	−0.18	−0.29 to −0.08	0.001	−0.17	−0.28 to −0.07	0.001
*Corynebacteriaceae*									
HRV, no symptoms	1.31	0.91 to 1.88	0.142	1.22	0.85 to 1.74	0.28	1.28	0.90 to 1.83	0.174
HRV, symptoms	0.91	0.64 to 1.30	0.611	1.04	0.73 to 1.48	0.828	1.1	0.78 to 1.55	0.588
*Moraxellaceae*									
HRV, no symptoms	0.81	0.58 to 1.12	0.208	0.91	0.65 to 1.27	0.584	0.89	0.64 to 1.24	0.49
HRV, symptoms	1.22	0.93 to 1.59	0.154	1.3	0.98 to 1.71	0.067	1.22	0.92 to 1.60	0.164
*Pasteurellaceae*									
HRV, no symptoms	0.85	0.55 to 1.32	0.476	0.94	0.60 to 1.48	0.801	0.97	0.61 to 1.53	0.895
HRV, symptoms	0.88	0.61 to 1.28	0.511	0.85	0.58 to 1.24	0.386	0.86	0.59 to 1.25	0.427
*Staphylococcaceae*									
HRV, no symptoms	1.37	0.94 to 2.01	0.103	1.2	0.82 to 1.76	0.348	1.24	0.84 to 1.83	0.282
HRV, symptoms	0.67	0.46 to 1.00	0.049	0.72	0.49 to 1.07	0.108	0.78	0.52 to 1.17	0.23
*Streptococcaceae*									
HRV, no symptoms	0.9	0.67 to 1.22	0.503	0.89	0.66 to 1.22	0.478	0.92	0.67 to 1.25	0.582
HRV, symptoms	0.8	0.61 to 1.05	0.111	0.81	0.62 to 1.07	0.135	0.86	0.65 to 1.13	0.272
*Carnobacteriaceae*									
HRV, no symptoms	1.21	0.80 to 1.85	0.37	1.14	0.74 to 1.77	0.543	1.15	0.75 to 1.78	0.522
HRV, symptoms	0.74	0.50 to 1.11	0.142	0.69	0.45 to 1.03	0.071	0.72	0.48 to 1.09	0.118
Others									
HRV, no symptoms	0.85	0.64 to 1.13	0.259	0.81	0.61 to 1.08	0.147	0.82	0.62 to 1.09	0.174
HRV, symptoms	0.7	0.54 to 0.90	0.006	0.66	0.51 to 0.85	0.001	0.7	0.54 to 0.90	0.006

a Analyses of the microbiota of samples with symptomatic and asymptomatic HRV colonization. Baseline samples are free of virus. Baseline (no virus in sample), *n* = 318; asymptomatic HRV infection, *n* = 51; symptomatic HRV infection, *n* = 69. Coinfections are not included.

b Adjusted for age and season.

c Adjusted for age, season, siblings, child care, breastfeeding, hypoallergenic nutrition, C-section, smoking in pregnancy, maternal atopy, parental education.

**FIG 2:**
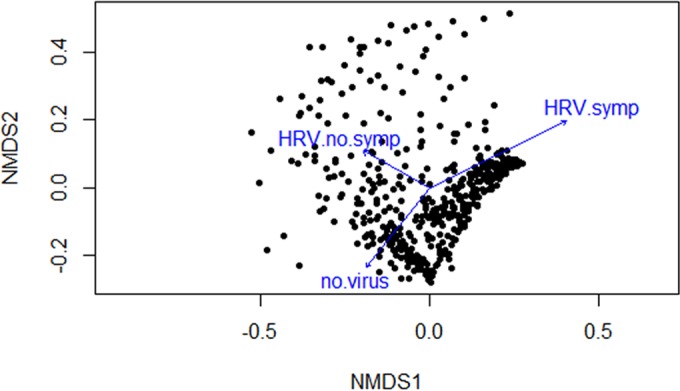
Beta diversity of the microbiota during HRV colonization. Shown is a comparison of the bacterial composition in samples without viral colonization to that in samples with asymptomatic and symptomatic HRV colonization. Weighted beta diversities are represented by using nonmetric multidimensional scaling (NMDS). Arrows indicate clustering of samples without viral colonization (no.virus), HRV colonization without symptoms (HRV.no.symp), and HRV infection with symptoms (HRV.symp). There is a significant difference in beta diversity during symptomatic HRV colonization (“Adonis” function of R; *P* = 0.04).

This change occurred only during periods of symptomatic HRV infections, whereas no change in density or diversity was found during asymptomatic HRV colonization (Table 3). In addition, using two different approaches (Jaccard and Bray-Curtis dissimilarities), there was a significantly different bacterial composition during symptomatic HRV infection (Jaccard, *P* = 0.04; Bray-Curtis, *P* = 0.034), but not during asymptomatic HRV colonization (Jaccard, *P* = 0.06; Bray-Curtis, *P* = 0.1) as revealed by a nonmetric multidimensional scaling (nMDS) ordination plot (Fig. 2).

To investigate if results were similar if only when including samples accompanied by more severe symptoms (cough and/or wheeze), we compared samples during HRV and upper respiratory tract infection (URTI) (*n* = 33), HRV colonization free of symptoms (*n* = 51), and samples free of virus and symptoms (*n* = 241) in a univariable model. Results were significant for bacterial density (PCR_concn_, incidence rate ratio [IRR], 1.53; 95% confidence interval [CI], 1.17 to 2.01; *P* = 0.002) and diversity (SDI coefficient [SDI coeff], −0.15; 95% CI, −0.30 to 0.00; *P* = 0.051), indicating comparable effects for mild and severe infections. To maintain higher statistical power, we combined both groups in our analysis.

Investigation on the bacterial family level showed that the relative abundance of the different bacterial families did not change during HRV colonization, but the abundance of “others” was significantly decreased (25% [SD, 36%] versus 31% [SD, 32%]) (Table 3; see Fig. S2 in the supplemental material).

10.1128/mSphere.00312-16.2 Figure S2  Abundances of bacterial families with and without HRV colonization. Comparison of the abundances (percentage) of the most frequent bacterial families in samples free of virus and free of symptoms, free of virus but with respiratory symptoms, during asymptomatic HRV colonization, and during symptomatic HRV colonization. Download Figure S2, PDF file, 0.1 MB. Copyright © 2016 Korten et al. 2016 Korten et al. This content is distributed under the terms of the Creative Commons Attribution 4.0 International license.

Findings were confirmed when samples with viral coinfections in the swab were included (results not shown) or in analyses adjusted for age and season and other risk factors (first and second groups of “Adjusted model” columns in Table 3, respectively). Results were verified in two sensitivity analyses: (i) excluding samples with a lower number of reads and (ii) if rarefied to 500 reads per sample (see Tables S2 and S3 in the supplemental material). To reassure that possible changes in the microbiota were due to viral colonization and were not the result of respiratory symptoms *per se*, we performed additional analyses, including samples free of virus with and without respiratory symptoms separately in the model (see Table S4 in the supplemental material). Results were not different from the calculations presented above, where samples free of virus irrespective of symptoms were used as the baseline. For further analysis, to obtain better statistical power, we thus referred to samples free of virus with and without respiratory symptoms as the baseline for comparison when investigating the microbiota during colonization of the different viruses.

10.1128/mSphere.00312-16.6 Table S2  Analysis of the association of HRV colonization with the microbiota in a subsample with a total number of reads of ≥500. Download Table S2, PDF file, 0.2 MB. Copyright © 2016 Korten et al. 2016 Korten et al. This content is distributed under the terms of the Creative Commons Attribution 4.0 International license.

10.1128/mSphere.00312-16.7 Table S3  Analysis of the association of HRV colonization with the microbiota in a subsample with the number of randomly resampled reads to 500. Download Table S3, PDF file, 0.2 MB. Copyright © 2016 Korten et al. 2016 Korten et al. This content is distributed under the terms of the Creative Commons Attribution 4.0 International license.

10.1128/mSphere.00312-16.8 Table S4  Unadjusted and adjusted analysis of the association of asymptomatic and symptomatic HRV infection with samples free of virus but from infants with respiratory symptoms. Download Table S4, PDF file, 0.1 MB. Copyright © 2016 Korten et al. 2016 Korten et al. This content is distributed under the terms of the Creative Commons Attribution 4.0 International license.

The presence of “viruses other than HRV” was not associated with changes in bacterial density (PCR_concn_), alpha diversity (SDI), or beta diversity (Jaccard dissimilarity) of the microbiota or any differences in the abundance of the bacterial families (see Table S5 in the supplemental material). We did not analyze associations of the different viruses separately, because occurrence was too rare for useful statistical analysis.

10.1128/mSphere.00312-16.9 Table S5  Unadjusted and adjusted analysis of the association of viruses “other than HRV” with the microbiota in the same sample. Download Table S5, PDF file, 0.1 MB. Copyright © 2016 Korten et al. 2016 Korten et al. This content is distributed under the terms of the Creative Commons Attribution 4.0 International license.

### Changes in the microbiota after viral colonization.

To investigate persistence of changes, we analyzed changes of the microbiota within 3 weeks after viral detection, as depicted in analysis II in Fig. 1. Because only symptomatic HRV colonization had shown an effect in analysis I, in this next step, we included in our analysis only cases of symptomatic HRV colonization and compared them to samples free of virus. Again we received significant differences in alpha diversity (SDI, 1.0 [SD, 0.7] versus 1.4 [SD, 0.9]), but the effect size was not as large (Table 4). Beta diversity, calculated by using nMDS, did not reach statistical significance, but trends were consistent (Jaccard, *P* = 0.1; Bray-Curtis, *P* = 0.12) (see Fig. S3A in the supplemental material). On the bacterial family level, the lower abundance of “others” remained (22% [24%] versus 33% [34%]), in addition we found a significantly higher abundance of *Moraxellaceae* (41.0% [36%] versus 28% [37%]) and a lower abundance of *Staphylococcaceae* (0.4% [0.8%] versus 5.4% [18.9%] after symptomatic HRV infections. For detailed results see Table 4. Results were confirmed in the two sensitivity analyses (results not shown).

10.1128/mSphere.00312-16.3 Figure S3  (A) Beta diversity of the microbiota within 3 weeks after viral colonization. Comparison of the bacterial composition within 3 weeks after symptomatic and asymptomatic HRV colonization, if the sample is than free of viral colonization and of two consecutive samples free of virus (the second sample). Weighted beta diversities are represented by using nMDS, grouped by HRV colonization without symptoms (HRV.no.symp), HRV colonization with symptoms (HRV.symp), and samples free of viral colonization (no.virus). Results are displayed in ellipses of standard deviation. There is no significant difference in beta diversities after symptomatic HRV colonization (“Adonis” function of R; *P* = 1). (B) Beta diversity of the microbiota within 3 weeks before viral colonization. Comparison of the bacterial composition in samples within 3 weeks before symptomatic and asymptomatic HRV colonization and samples remaining free of viral colonization (two consecutive samples free of virus and the first sample). Weighted beta diversities are represented by using nMDS, grouped by HRV colonization without symptoms (HRV.no.symp), HRV colonization with symptoms (HRV.symp), and samples free of viral colonization (no.virus). Results are displayed in ellipses of standard deviation. There is no significant difference in beta diversities between the three groups (“Adonis” function of R). Download Figure S3, PDF file, 0.1 MB. Copyright © 2016 Korten et al. 2016 Korten et al. This content is distributed under the terms of the Creative Commons Attribution 4.0 International license.

**TABLE 4:** Unadjusted and adjusted analysis of the association of symptomatic HRV colonization with the microbiota within 3 weeks after HRV colonization^a^

Outcome	Unadjusted model			1st adjusted model^b^			2nd adjusted model^c^		
	IRR/Coeff	95% CI	*P*	IRR/Coeff	95% CI	*P*	IRR/Coeff	95% CI	*P*
PCR_concn_	1.4	1.10 to 1.78	0.007	1.3	1.02 to 1.67	0.035	1.24	0.97 to 1.58	0.092
SDI	−0.1	−0.23 to 0.03	0.124	−0.14	−0.27 to −0.01	0.035	−0.13	−0.26 to 0.00	0.05
*Corynebacteriaceae*	1.02	0.67 to 1.54	0.942	1.04	0.68 to 1.58	0.86	1	0.66 to 1.51	0.996
*Moraxellaceae*	1.68	1.21 to 2.32	0.002	1.74	1.23 to 2.46	0.002	1.48	1.04 to 2.10	0.03
*Pasteurellaceae*	0.94	0.59 to 1.51	0.807	0.85	0.53 to 1.38	0.521	0.84	0.52 to 1.36	0.483
*Staphylococcaceae*	0.57	0.35 to 0.94	0.028	0.54	0.32 to 0.92	0.022	0.54	0.32 to 0.93	0.026
*Streptococcaceae*	0.98	0.71 to 1.37	0.927	0.91	0.65 to 1.27	0.573	0.93	0.66 to 1.30	0.656
*Carnobacteriaceae*	0.86	0.54 to 1.37	0.527	0.82	0.51 to 1.32	0.414	0.84	0.52 to 1.35	0.472
Others	0.71	0.52 to 0.98	0.039	0.69	0.49 to 0.95	0.026	0.71	0.51 to 0.99	0.044

a Analysis of the microbiota ≤3 weeks after symptomatic HRV colonization, if then free of symptomatic HRV compared to samples that had no virus ≤3 weeks before. Baseline (no virus in sample), *n* = 256; symptomatic HRV infection, *n* = 42. Coinfections are not included.

b Adjusted for age and season.

c Adjusted for age, season, siblings, child care, breastfeeding, hypoallergenic nutrition, C-section, smoking in pregnancy, maternal atopy, and parental education.

**FIG 3:**
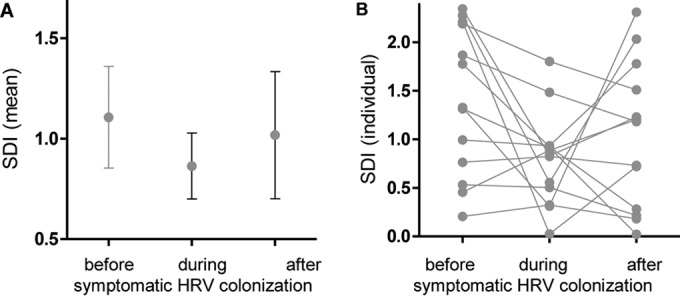
SDI values before, during, and after symptomatic HRV colonization. (A) Mean (95% CI) of SDI before, during, and after symptomatic HRV colonization. All samples are included. (B) Individual values of SDI before, during, and after symptomatic HRV colonization. Presented are only swabs with the following characteristics: three consecutive samples with the one before HRV colonization free of any viral colonization, the sample during HRV colonization without coinfection, and the sample after HRV colonization free of any viral colonization.

Although changes in the microbiota during symptomatic HRV infections were highly significant if pooled (Fig. 3A), individual courses showed a heterogeneous picture (Fig. 3B), indicating large interindividual differences and suggesting that a personalized microbiota in each individual exists.

### Microbiota as a factor influencing subsequent HRV colonization.

To assess whether microbiota influences the occurrence and/or severity of subsequent viral infections, as shown in analysis III in Fig. 1, we investigated the association of preexisting microbial composition on viral colonization within the following 3 weeks. Neither alpha diversity nor beta diversity (Fig. S3B), bacterial density, nor the abundances of the bacterial families were associated with a higher prevalence of HRV colonization at the following swab. Furthermore, we found that none of the analyzed microbiota outcome parameters had an influence on whether or not subsequent HRV colonization was accompanied by respiratory symptoms (see Table S6 in the supplemental material).

10.1128/mSphere.00312-16.10 Table S6  Unadjusted and adjusted analysis of the association of the microbiota within 3 weeks before symptomatic and asymptomatic HRV infection. Download Table S6, PDF file, 0.1 MB. Copyright © 2016 Korten et al. 2016 Korten et al. This content is distributed under the terms of the Creative Commons Attribution 4.0 International license.

### Persistent changes of the microbiota after frequent HRV colonization.

To assess long-term consequences, we evaluated the overall influence of frequent HRV colonization on the microbiota of the last 3 months of the study. The mean number of samples per child was 6 (range, 4 to 8) in 31 infants, as one infant stopped the study at 8 months of age.

**FIG 4:**
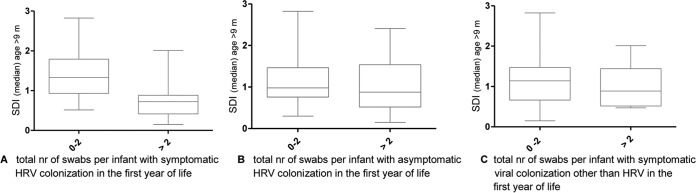
SDI at the end of the first year of life in relation to the number of viral colonizations throughout the whole year. Shown are the median SDIs of the nasal swabs taken in the last 3 months of the study period (box plots with minimum to maximum) grouped as from zero to two positive swabs and as more than two positive swabs for (A) symptomatic HRV (0 to 2, *n* = 18; >2, *n* = 13), (B) asymptomatic HRV (0 to 2, *n* = 20; >2, *n* = 11), and (C) symptomatic viral infections other than HRV (0 to 2, *n* = 21; >2, *n* = 10). Coinfections are only included for symptomatic viral infections other than HRV. A lower SDI was found in infants with more frequent symptomatic HRV colonization (*P* = 0.015) but not with more frequent asymptomatic HRV colonization (*P* = 0.2) or more frequent symptomatic viral colonization other than HRV (*P* = 0.1).

More frequent symptomatic HRV infections during the first year of life were associated with a lower SDI at the end of the study period (SDI coeff, −0.5; 95% CI, −1.0 to −0.01; *P* = 0.042) (Fig. 4A). This was not found in infants with more frequent asymptomatic HRV colonization (SDI coeff, −0.2; 95% CI, −0.7 to 0.3; *P* = 0.4) or more frequent symptomatic infections of “viruses other than HRV” (SDI coeff, 0.04; 95% CI, −0.6 to 0.7; *P* = 0.9) (Fig. 4B and C). Changes were not due to differences of SDI at baseline, as baseline SDI values were comparable between different groups at the age of 3 months or younger (data not shown). Findings were robust if coinfections were included and showed a linear association (see Fig. S4 in the supplemental material).

10.1128/mSphere.00312-16.4 Figure S4  SDI at the end of the study period in relation to the number of HRV infections throughout the whole year. Shown is the median SDI of the nasal swabs taken in the last 3 months of the study period (age 9 months or older) for each infant, classified by the numbers of positive swabs for symptomatic HRV colonization during the first year of life. Coinfections are included. A higher number of positive swabs is associated with a lower SDI (*P* = 0.01). Download Figure S4, PDF file, 0.1 MB. Copyright © 2016 Korten et al. 2016 Korten et al. This content is distributed under the terms of the Creative Commons Attribution 4.0 International license.

## DISCUSSION

Our study is the first longitudinal study describing the interaction of viral colonization with the upper respiratory tract microbiota in the first year of life using such a dense sampling frequency. The weekly monitoring of respiratory symptoms enabled us to distinguish between symptomatic and asymptomatic episodes of viral colonization. Analyzing 559 biweekly nasal samples, we found a strong and robust association between HRV colonization and changes in the microbiota. During symptomatic HRV colonization, a lower alpha diversity (SDI), a higher bacterial density (PCR_concn_), and a difference in beta diversity (Jaccard and Bray-Curtis dissimilarities) of the microbiota were found. In contrast, during asymptomatic HRV colonization, no changes in the microbiota were detected. Results remained stable if distinguishing between symptomatic and asymptomatic samples free of virus, indicating that changes in the microbiota are not driven by symptoms *per se* but by HRV infection. Furthermore, in addition to the short-term changes, more frequent symptomatic HRV infections had a sustained impact upon the composition of the microbiota.

To our knowledge, an investigation of viral colonization of the nose and the nasal microbiota in infancy has only been published once (23). In that Australian study, sampling was performed two or three times in each infant, so persisting changes were not investigated. The authors concluded that viruses and the microbiota contribute independently to the later onset of asthma, but the underlying interactions between virus and bacteria were not assessed on a short-term basis. Apart from that study, the impact of symptomatic HRV infection on bacterial colonization has been reported only in older children (24). The authors reported a higher detection rate and a higher quantity of bacteria during HRV infection in children of ages 4 to 12 years, as well as an association of specific bacteria and HRV infection with asthma symptoms.

As reported before from adult studies, we found a lower diversity and a higher density during symptomatic HRV colonization (20, 25, 26). The rather high intra- and interindividual heterogeneity is also in line with previous data (10, 25) and supports the hypothesis of a different composition of the microbiota during viral infection (27), possibly explained by an outgrowth of potential pathogens triggered through HRV.

One drawback of existing studies investigating the microbiota and viral infections is the lack of including samples during asymptomatic periods of viral colonization, as information about asymptomatic HRV colonization in infancy is scarce in general (13, 28, 29). This is, however, needed if one aims to differentiate between asymptomatic HRV colonization and HRV infection. In our study, only symptomatic HRV infections were associated with changes in the microbiota. Importantly, we could also show that respiratory symptoms alone did not have the effect on the composition of the microbiota, indicating that a certain immune response of the individual subject driven by viruses is needed to result in changes of the microbiota (30).

The changes in the microbiota did partly persist 3 weeks after viral colonization, although the association was less strong. Studies in adults reported no sustained changes in healthy subjects (20), whereas in chronic obstructive pulmonary disease (COPD) patients, a higher abundance of *Haemophilus influenzae* and an increase in 16S rRNA gene copy number could be seen up to 42 days after infection (20). Our finding of a higher abundance of *Moraxellaceae* and a lower abundance of *Staphylococcaceae* after HRV infection was not reported before, but other studies described an association of a higher abundance of *Moraxellaceae* with acute respiratory infections (ARIs) (23) and viral colonization (26). These results support the hypothesis of persistent bacterial outgrowth after viral infection, especially as *Moraxella catarrhalis* is a potential pathogen in respiratory disease (1).

Similar to previous data from adults, in our study, the microbiota composition was no risk factor for subsequent viral colonization and had no impact upon occurrence or severity of HRV infections (25). This suggests an influence of the virus on the microbiota and not vice versa; however, to clearly examine this, “virus-naïve” samples should be assessed in future studies.

The lower SDI at the end of the study period in infants with more frequent symptomatic HRV further suggests continuing changes in the microbiota and is in line with recent results (23), representing a speculative but possible link to later disease development. It is yet unclear if it is the frequency of infections, the time period during which they occur, or preexisting susceptibility that is most important for persisting changes in the microbiota. In addition, although the difference in alpha diversity was highly significant, rather high inter- and intraindividual standard deviations have to be kept in mind before considering it as potential clinical marker. However, if confirmed in other studies, alpha diversity could be used as an easy outcome parameter to assess individual susceptibility toward ongoing microbial alterations upon viral infections and potentially later disease development.

A major strength of our study is the dense sampling within the first year of life. Furthermore, data assessment was not restricted to periods of illness or scheduled visits. Our study provides longitudinal data on the dynamic interplay between virus and the microbiota in the first year of life, considering 12 different viruses. Due to rare occurrence, we could not obtain clear results for all viruses; however, with the high number of different viruses analyzed, we can differentiate between samples free of virus, those free of viral coinfection, and those with viral infection; the weekly information and longitudinal sampling further allowed us to assess associations in both directions with different time lags. Having detailed information of pre-, peri-, and postnatal history and exact documentation of any changes of sociodemographic and environmental factors, including family history for atopy, we were able to adjust for several known confounders and other risk factors, ensuring results are robust.

A limitation is the small number of study infants. Despite the high number of samples, a larger study population is needed to detect additional differences between subjects. Importantly, analysis of viruses and the microbiota during respiratory infections in a higher number of infants is needed to understand the role of viruses other than HRV. Also, the causal and mechanistic pathways between symptomatic HRV infections and the microbiota remain unclear: these need to be assessed in translational approaches or using animal models.

Viral and bacterial colonization are thought to be causal in acute respiratory morbidity, as well as in the later development of chronic diseases, such as asthma or CF (7, 14, 20). Especially HRV infections drive specific and nonspecific immune reactions and inflammation (31) through dendritic cells, macrophages, and epithelial cells and a number of cytokines (32–34). If future studies including sampling of local or systemic inflammatory processes (35, 36) can show that those mechanisms play an important role in viral colonization during infancy and in shaping the microbiota-immune balance, preventative measures, such as vaccination or application of probiotics, could be directed against those processes. Although further studies are clearly needed to confirm this hypothesis, the first studies have already shown a possibly preventive effect of probiotics on respiratory infection in general and HRV infection in particular (37, 38).

We conclude that symptomatic HRV infections are associated with a short-term change in bacterial density and diversity of the microbiota and that more frequent symptomatic HRV infections have a long-term impact upon the diversity of the microbiota at the end of the first year of life. This indicates a dynamic interaction between HRV infections and the respiratory microbiota in early life. Although the long-term importance of these interactions needs to be examined in future follow-up studies, our findings could be of significance for preventive or therapeutic procedures.

## MATERIALS AND METHODS

### Study design and subjects.

Thirty-two healthy, unselected infants from the ongoing prospective Basel-Bern Infant Lung Development (BILD) cohort study were included between April 2010 and February 2013 (39). Pregnant mothers were recruited at maternity hospitals and practices of obstetricians by advertisements and interviews. Exclusion criteria for the BILD cohort study and thus our subgroup analysis are ethnicity other than white, preterm delivery (<37 weeks), major birth defects, disease or later diagnosis of airway malformation, or specific chronic respiratory disease.

A weekly telephone interview was performed by study nurses to assess respiratory health. This substudy was performed in Bern only and thus approved by the Ethics Committee Bern.

### Nasal swab procedure.

An anterior nasal swab (FLOQSwabs, in room temperature universal transport medium [UTM-RT]; Copan, Italia) was collected biweekly by parents after being instructed by study nurses about correct and standardized sampling of the swabs, starting in the fifth week of life. Immediately after acquisition, nasal swabs were sent to our study center and frozen at −80°C. The microbiota, 10 different viruses, and two atypical bacteria were analyzed in each sample.

### Microbiota analysis.

The methods used for PCR amplification of the 16S ribosomal RNA (rRNA) gene and 454 amplicon sequencing are described in detail elsewhere (10, 40). In brief, V3 to V5 regions of the bacterial 16S rRNA genes were amplified using the primer pair 341F/907R. The following primers were used: 341F (5′-*CGTATCGCCTCCCTCGCGCCA*TCAGXXXXXXXXXX**ACTCCTACGGGAGGCAGCAG**-3′) and 907R (5′-*CTATGCGCCTTGCCAGCCCGC*TCAGXXXXXXXXXX**CCGTCAATTCMTTTGAGTTT**-3′). The template-specific sequences are in boldface, adaptor sequences are italicized, and the XXXXXXXXXX sequences describe the sample-specific multiplex identifier (MID) tag barcode. PCR mixtures were eluted in 40 µl of double-distilled water, after purification with the Wizard SV PCR cleanup system (Promega, Madison, WI). PCR products with a concentration of less than 1.0 ng/µl were excluded from the study, corresponding to less than 1 pg/µl bacterial DNA (10), which was recently recommended as a reliable threshold (41). For further quality control, we excluded samples with less than 70 reads and sequenced two negative-control samples. Samples displaying greater than 5% sequence reads identical to the negative controls were excluded. In addition, we did not identify taxa that have recently been identified to be contaminations (42). A 40-ng/µl concentration of each PCR product was pooled, and every MID was used once. The 8 resulting amplicon pools were sequenced with the 454 sequencing platform, and the reads were submitted to the National Center for Biotechnology Information Sequence Read Archive. Analysis of sequencing products was performed using PyroTagger pipeline (43), which comprises the definition of operational taxonomic units (OTUs) based on 97% sequencing identity, estimation of chimeras, and taxonomic assignments. Pipeline settings were described in detail before (40). As an additional quality cutoff, samples with less than 70 sequence reads were excluded from the study (*n* = 13), resulting in a minimum number of reads of 77. The mean number of sequence reads was, however, much higher (1,469 [SD, 1,083] reads per sample).

Outcome parameters were bacterial density estimated by the concentration of the 16S rRNA PCR product (PCR_concn_) in nanograms per microliter, alpha diversity assessed by the Shannon diversity index (SDI, based on OTUs of 97% sequence identity), and beta diversity, the latter both calculated in R using the “vegan” package. For beta diversity, dissimilarity indices were calculated using the Jaccard distance method and the Bray-Curtis dissimilarity, and nMDS was used as the ordination method. Again, beta diversity measurements were based on OTUs of 97% sequence identity.

The five most abundant bacterial families (*Streptococcaceae*, *Staphylococcaceae*, *Moraxellaceae*, *Corynebacteriaceae*, and *Pasteurellaceae*) and *Carnobacteriaceae* (which were formerly described in interrelationship with respiratory infections [23]) were analyzed, and the remaining families were grouped as “others.” This phylogenetic level was used for additional statistical analyses (see below).

### Virological analysis.

The following different viruses and atypical bacteria were used as outcome parameters in our analysis: influenza A and influenza B viruses, RSV, human metapneumovirus (hMPV), adenovirus (ADV), human Bocavirus (hBoV), rhinovirus/enterovirus (HRV/EV), parechovirus, coronavirus (HCoV), parainfluenzavirus (HPIV), and *Mycoplasma pneumoniae* and *Chlamydia pneumoniae*.

The samples were tested by real-time PCR, using a combination from the 7-duplex Respiratory multiwell system (MWS) r-gene (influenza virus A/B, RSV/hMPV, rhinovirus and EV/cellular gene control [CC], ADV/hBoV, HCoV/HPIV, *C. pneumoniae/M. pneumoniae*, and parechovirus) commercialized by Argene/BioMérieux (Marcy l’Etoile, France), according to the manufacturer’s instructions. RNA and DNA were extracted from a 400-µl sample with the NucliSENS easyMAG (bioMérieux, Marcy l’Etoile, France) according to the manufacturer’s instructions and eluted in 110 µl.

The real-time PCR was performed on different real-time PCR machines of Applied Biosystems (7500, 7900HT; QuantStudio 7 Flex).

The hypoxanthine phosphoribosyltransferase 1 (HPRT1) cellular gene control (CC) essay using the duplex rhinovirus and EV/CC r-gene samples was used to evaluate sampling quality, extraction, and amplification in every sample. Samples showing an exponential amplification curve with a threshold cycle (*C*_*T*_) value of <45 were assessed as positive.

The PCR tests were done by a certified laboratory that has routinely used the CE-marked Respiratory MWS r-gene for the detection of respiratory viruses since 2012.

For easier reading, the term “viral analysis” is used in the text, although two atypical bacteria are also included in the analysis.

### Respiratory symptoms.

In weekly standardized telephone interviews, symptoms of lower and upper respiratory tract infections, wheeze, and/or cough were recorded. Rhinitis (runny or blocked nose) was independently assessed as the most common upper respiratory symptom. For data analysis, if referring to symptomatic viral infection, we combined upper and lower respiratory symptoms. If referring to URTI, cough and/or wheeze had to be present. An LRTI was defined as cough, wheeze, or breathing difficulties and upper respiratory tract symptoms or elevated temperature for more than 2 consecutive days. A viral infection was specified as symptomatic if any respiratory symptoms occurred up to 4 days before until 7 days after detection of viral colonization in the sample.

### Additional risk factors or confounders.

Information about pre-, peri-, and postnatal history, including family, maternal, sociodemographic, and environmental histories, was collected in hospital records and with a questionnaire. At the time of the weekly standardized phone interview, changes in host and environmental factors were documented. The following factors were included in our statistical model to adjust for potential confounders: the time-invariant factors included in our study were maternal atopic disease (maternal asthma, hay fever, or eczema), parental education (categorized as low [less than 4 years of secondary education], middle [at least 4 years of secondary education], and high [tertiary education]), mode of delivery (C-section or vaginal delivery), smoking during pregnancy, and presence of older siblings. The time-variant factors included were age and season of sampling, breastfeeding (“current” [yes/no] at time of swab), hypoallergenic nutrition (“current” [yes/no] at time of swab), and child care (“current” [yes/no] at time of swab).

### Statistical analysis.

We investigated the association between viral colonization and the microbiota using multilevel regression with random effect to correct for clustering on the individual level. We performed binomial multilevel regression to analyze bacterial families and PCR_concn_. In a subgroup analysis, zero inflated binomial regression was not a better statistical model than regular binomial regression (Vuong test for model selection) and thus was not used. Diversity (SDI) was analyzed with multilevel linear regression after transformation (square root [sqrt]) to obtain a normal distribution.

We investigated an association between viral colonization and the microbiota in the same sample (Fig. 1; analysis I), the microbiota after viral colonization (Fig. 1; analysis II), and the microbiota before viral colonization (Fig. 1; analysis III).

After performing a univariable model (no adjustment), two adjusted models were performed. In the first adjusted model, an adjustment for season and age was performed, because both influence the microbiota (10). In the second adjusted model, we adjusted for further risk factors of respiratory symptoms or viral infections in early life: having siblings, mode of delivery, child care, hypoallergenic nutrition, breastfeeding, parental education, smoking during pregnancy, and maternal atopy (44–48). To analyze the microbiota at the end of the study period, we calculated the median of the different microbiota outcome parameters of all existing samples of an infant between 9 and 12 months of age.

We used two different indices to calculate the diversity between samples. The Bray-Curtis dissimilarity and the Manhattan-type Jaccard dissimilarity were used to calculate the weighted beta diversity indices (abundance based), by using the “vegdist” function of the “vegan” package in R. For nMDS, “metaMDS” of the vegan package was used. To analyze differences in beta diversity, the “Adonis function” was used as a permutational multivariate analysis of variance (PERMANOVA) analysis, and adjustment for multiple measurements was applied (“strata” operator in the Adonis function).

We performed two sensitivity analyses to ensure robustness of results. We excluded the samples with a number of reads below 500 (*n* = 84) and (i) repeated the main analyses as stated above. With this approach the range of number of sequences per sample between the different samples is much smaller. (ii) The total number of sample reads was randomly resampled to an equal number of reads and rarefied to 500, using the “rrarefy” command of the vegan package in R. Subsample analysis in all remaining samples with 500 reads (*n* = 475) was performed.

Results are represented as coefficients (Coeff), incidence rate ratios (IRR), or odds ratios (OR) with 95% confidence intervals (CIs) and *P* values; *P* values of <0.05 were considered statistically significant. All analyses were done using Stata13 (College Station, TX), R (“vegan package” “Adonis function”) version 3.1.1 (R Foundation of Statistical Computing), and GraphPad Prism 5.

### Accession number(s).

Reads of this study are part of the sequencing data deposited in NCBI’s Sequence Read Archive under GenBank accession no. SRP041616.
