# Supplementary figures and images for: Interactions of Respiratory Viruses and the Nasal Microbiota during the First Year of Life in Healthy Infants
Source: mSphere. 2016 Nov 23;1(6):e00312-16. doi: 10.1128/mSphere.00312-16 (PMC5120172; doi:10.1128/mSphere.00312-16)

**Figure S1**

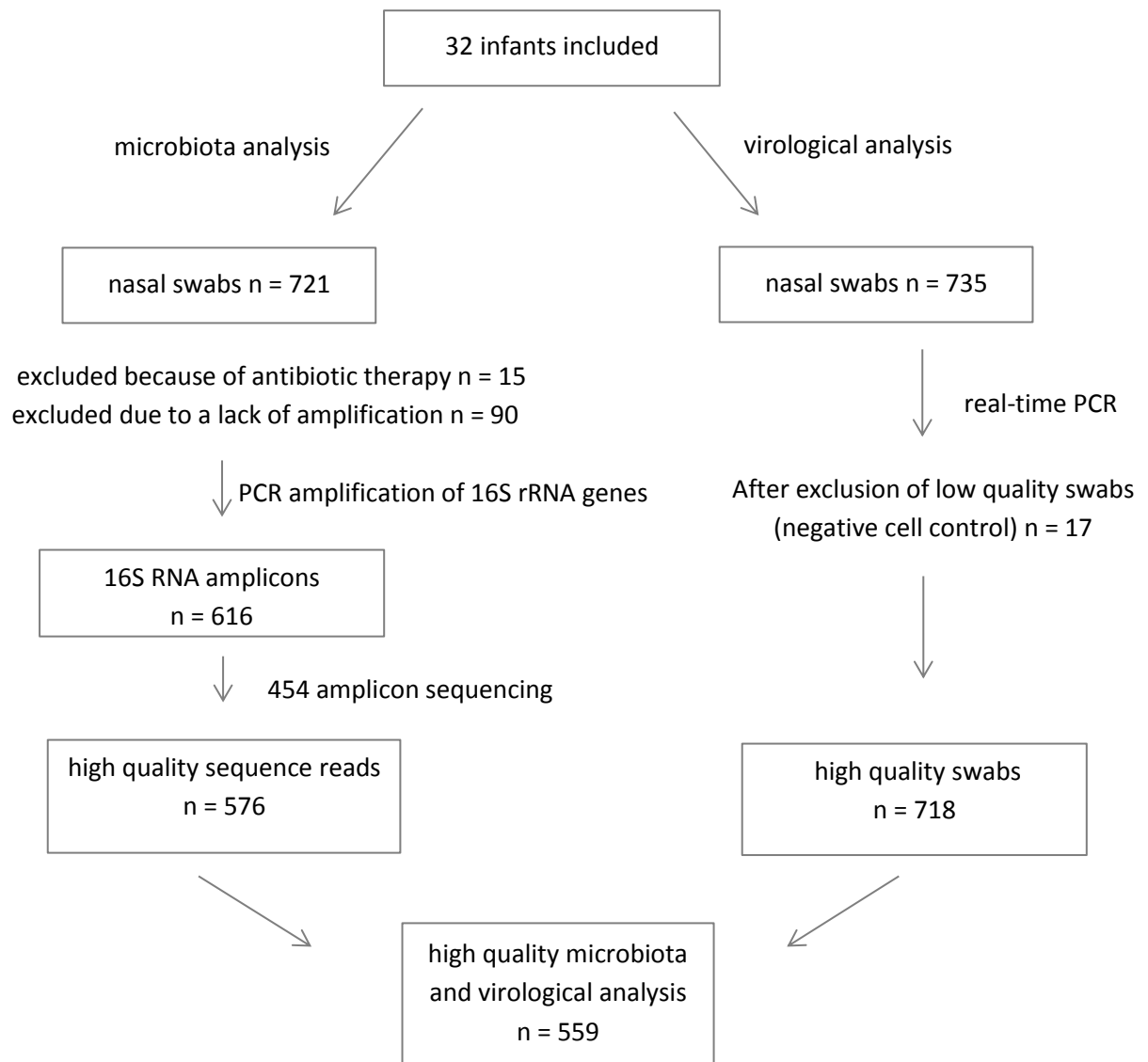

Supplement: Figure S1 [file sph006162193sf7.pdf]

**Figure S2**

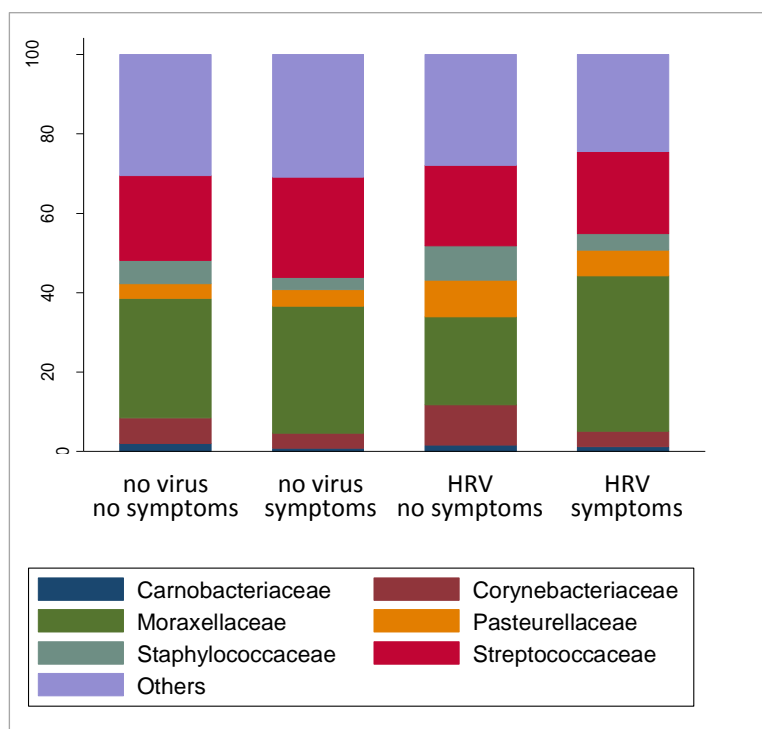

Supplement: Figure S2 [file sph006162193sf8.pdf]

Figure S3

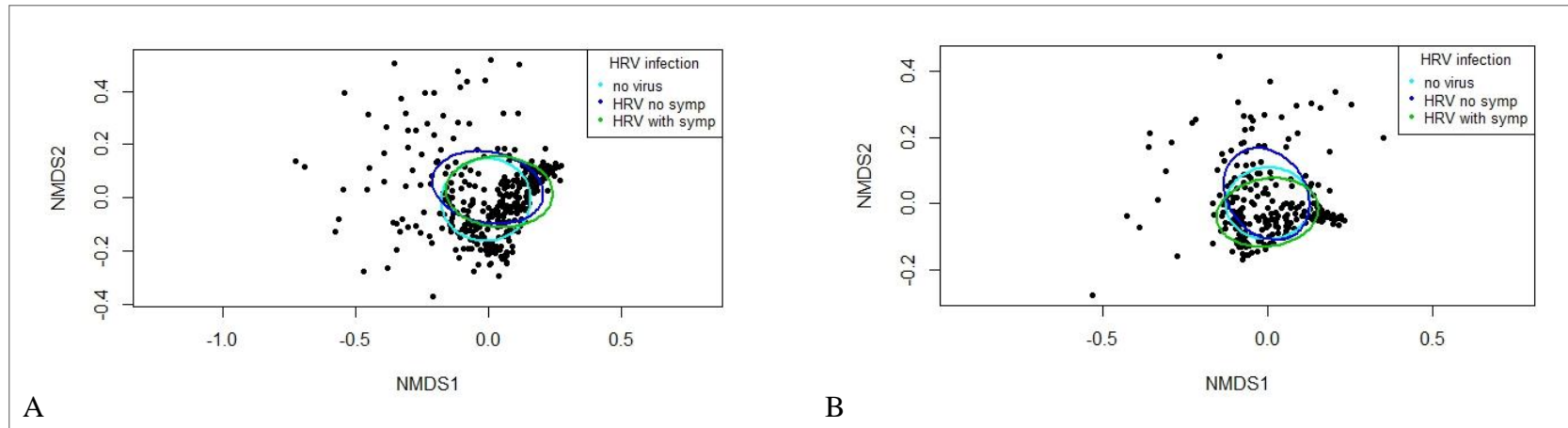

Supplement: Figure S3 [file sph006162193sf9.pdf]

**Figure S4**

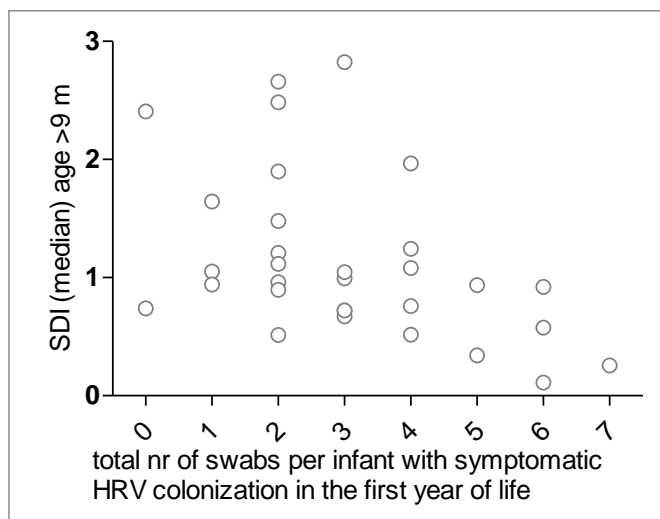

Supplement: Figure S4 [file sph006162193sf10.pdf]
