# Supplementary material for: Interactions of Respiratory Viruses and the Nasal Microbiota during the First Year of Life in Healthy Infants
Source: mSphere. 2016 Nov 23;1(6):e00312-16. doi: 10.1128/mSphere.00312-16 (PMC5120172; doi:10.1128/mSphere.00312-16)
Supplement: Table S1 [file sph006162193st1.pdf]

Table S1: Risk factors for HRV colonization

Characteristic			
Pregnancy:	Smoking in pregnancy, no. (%)		3 (9)
Birth:	C-section, no.(%)		7 (22)
Nutrition:	Breast-feeding, no. (%)		32 (100)
	Hypoallergenic nutrition*, no. (%)		8 (25)
Family history:	Maternal atopy, no (%)		9 (28)
Environment:	Siblings, no. (%)	0	7 (22)
		1	15 (47)
		>=2	10 (33)
	Childcare**, no. (%)		7 (22)
Parental education***:		0	3 (9)
		1	13 (41)
		2	16 (50)

n = 32 infants
*hypoallergenic nutrition is defined as hypoallergenic milk supplements in the first year of life at any timepoint
**childcare is defined as attending childcare in the first year of life at any timepoint.
***parental education is categorized into low (0, less than four years of apprenticeship), middle (1, at least four years of apprenticeship) and high (2, tertiary education).
