# Supplementary material for: Interactions of Respiratory Viruses and the Nasal Microbiota during the First Year of Life in Healthy Infants
Source: mSphere. 2016 Nov 23;1(6):e00312-16. doi: 10.1128/mSphere.00312-16 (PMC5120172; doi:10.1128/mSphere.00312-16)
Supplement: Table S2 [file sph006162193st2.pdf]

Table S2: Analysis of the association of HRV colonization with the microbiota in a subsample with total nr. of reads >=500

	unadjusted model			adjusted model[a]			adjusted model[b]		
Outcome	IRR/ Coef	95% CI	p	IRR/ Coef	95% CI	p	IRR/ Coef	95% CI	p
PCRconc									
HRV – no symptoms	1.14	[0.88,1.48]	0.32	1.22	[0.94,1.59]	0.14	1.18	[0.90,1.54]	0.23
HRV – plus symptoms	1.44	[1.17,1.78]	0	1.43	[1.16,1.76]	0	1.33	[1.08,1.64]	0.01
SDI									
HRV – no symptoms	0.05	[-0.08,0.18]	0.481	0	[-0.13,0.13]	0.974	0	[-0.13,0.13]	0.99
HRV – plus symptoms	-0.18	[-0.28,-0.07]	0.001	-0.19	[-0.30,-0.08]	0.001	-0.18	[-0.29,-0.07]	0.001
Corynebacteriaceae									
HRV – no symptoms	1.3	[0.85,2.00]	0.232	1.21	[0.78,1.86]	0.391	1.18	[0.77,1.80]	0.448
HRV – plus symptoms	1.02	[0.68,1.51]	0.94	1.06	[0.71,1.56]	0.787	1.05	[0.71,1.54]	0.809
Moraxellaceae									
HRV – no symptoms	0.86	[0.60,1.23]	0.421	1.03	[0.71,1.49]	0.892	1.06	[0.74,1.51]	0.766
HRV – plus symptoms	1.18	[0.89,1.58]	0.249	1.32	[0.98,1.77]	0.065	1.23	[0.92,1.65]	0.154
Pasteurellaceae									
HRV – no symptoms	0.99	[0.61,1.60]	0.962	1.11	[0.68,1.82]	0.674	1.12	[0.68,1.85]	0.661
HRV – plus symptoms	0.91	[0.61,1.35]	0.633	0.87	[0.58,1.31]	0.512	0.89	[0.59,1.33]	0.565
Staphylococcaceae									
HRV – no symptoms	1.27	[0.82,1.97]	0.291	1.03	[0.66,1.63]	0.889	1.05	[0.66,1.69]	0.823
HRV – plus symptoms	0.7	[0.46,1.08]	0.109	0.73	[0.47,1.12]	0.148	0.74	[0.48,1.15]	0.184
Others									

HRV – no symptoms	0.84	[0.61,1.15]	0.279	0.77	[0.56,1.07]	0.123	0.77	[0.56,1.06]	0.103
HRV – plus symptoms	0.68	[0.52,0.90]	0.007	0.62	[0.47,0.82]	0.001	0.66	[0.50,0.86]	0.003

Analyses of the microbiota of samples with symptomatic and asymptomatic HRV colonization in a subsample including only samples with >= 500 reads. Displayed are relative abundances. Baseline are samples free of virus.

Baseline: no virus in sample (n = 262); asymptomatic HRV infection (n = 41); symptomatic HRV infection (n=63); co-infections are not included; [a]adjusted for age and season; [b]adjusted for age, season, siblings, childcare, breastfeeding, hypoallergenic nutrition, C-section, smoking in pregnancy, maternal atopy, parental education
