# Supplementary material for: Interactions of Respiratory Viruses and the Nasal Microbiota during the First Year of Life in Healthy Infants
Source: mSphere. 2016 Nov 23;1(6):e00312-16. doi: 10.1128/mSphere.00312-16 (PMC5120172; doi:10.1128/mSphere.00312-16)
Supplement: Table S3 [file sph006162193st3.pdf]

Table S3: Analysis of the association of HRV colonization with the microbiota in a subsample with randomly resampled reads to 500

	unadjusted model			adjusted model[a]			adjusted model[b]		
Outcome	IRR/ Coef	95% CI	p	IRR/ Coef	95% CI	p	IRR/ Coef	95% CI	p
PCRconc									
HRV – no symptoms	1.15	[0.88,1.49]	0.31	1.23	[0.94,1.60]	0.13	1.18	[0.90,1.54]	0.23
HRV – plus symptoms	1.43	[1.17,1.77]	<0.001	1.41	[1.15,1.74]	0	1.32	[1.07,1.62]	0.01
SDI									
HRV – no symptoms	0.05	[-0.08,0.18]	0.415	0	[-0.13,0.14]	0.943	0.01	[-0.13,0.14]	0.909
HRV – plus symptoms	-0.16	[-0.27,-0.05]	0.004	-0.17	[-0.28,-0.07]	0.002	-0.16	[-0.27,-0.05]	0.004
Corynebacteriaceae									
HRV – no symptoms	1.41	[0.81,2.46]	0.221	1.21	[0.69,2.14]	0.503	1.29	[0.73,2.30]	0.38
HRV – plus symptoms	0.69	[0.41,1.19]	0.183	0.64	[0.38,1.11]	0.111	0.66	[0.38,1.15]	0.144
Moraxellaceae									
HRV – no symptoms	0.95	[0.67,1.36]	0.78	1.1	[0.76,1.59]	0.625	1.13	[0.78,1.63]	0.519
HRV – plus symptoms	1.26	[0.94,1.67]	0.12	1.39	[1.03,1.87]	0.031	1.31	[0.97,1.77]	0.074
Pasteurellaceae									
HRV – no symptoms	1.09	[0.69,1.71]	0.716	1.19	[0.75,1.91]	0.455	1.23	[0.77,1.97]	0.395
HRV – plus symptoms	0.93	[0.63,1.37]	0.706	0.89	[0.60,1.31]	0.553	0.92	[0.62,1.37]	0.689
Others									
HRV – no symptoms	0.89	[0.66,1.20]	0.453	0.81	[0.59,1.10]	0.17	0.81	[0.60,1.10]	0.184
HRV – plus symptoms	0.75	[0.58,0.97]	0.029	0.68	[0.52,0.88]	0.004	0.7	[0.54,0.91]	0.009

Analyses of the microbiota of samples with symptomatic and asymptomatic HRV colonization in a subsample with total number of sample reads randomly resampled to 500. Displayed are total nr. of reads of the bacterial families. Baseline are samples free of virus. Baseline: no virus in sample (n = 262); asymptomatic HRV infection (n = 41); symptomatic HRV infection (n=63); co-infections are not included; [a]adjusted for age and season; [b]adjusted for age, season, siblings, childcare, breastfeeding, hypoallergenic nutrition, C-section, smoking in pregnancy, maternal atopy, parental education
