# Supplementary material for: Interactions of Respiratory Viruses and the Nasal Microbiota during the First Year of Life in Healthy Infants
Source: mSphere. 2016 Nov 23;1(6):e00312-16. doi: 10.1128/mSphere.00312-16 (PMC5120172; doi:10.1128/mSphere.00312-16)
Supplement: Table S4 [file sph006162193st4.pdf]

Table S4: unadjusted and adjusted analysis of the association of asymptomatic and symptomatic HRV infection with samples free of virus but respiratory symptoms

	unadjusted model			adjusted model[a]			adjusted model[b]		
Outcome	IRR/ Coef	95% CI	p	IRR/ Coef	95% CI	p	IRR/ Coef	95% CI	p
PCRconc									
no virus – plus symptoms	1.11	[0.90,1.37]	0.32	1.06	[0.86,1.30]	0.61	1.02	[0.83,1.26]	0.83
HRV – no symptoms	1.15	[0.91,1.47]	0.25	1.19	[0.94,1.51]	0.15	1.15	[0.91,1.47]	0.24
HRV – plus symptoms	1.49	[1.21,1.83]	<0.001	1.41	[1.15,1.73]	<0.001	1.31	[1.07,1.61]	0.01
SDI									
no virus – plus symptoms	-0.05	[-0.15,0.05]	0.335	-0.03	[-0.13,0.08]	0.612	-0.03	[-0.13,0.08]	0.583
HRV – no symptoms	0.04	[-0.08,0.16]	0.503	0.02	[-0.10,0.14]	0.778	0.02	[-0.10,0.14]	0.771
HRV – plus symptoms	-0.19	[-0.30,-0.08]	0.001	-0.19	[-0.30,-0.08]	0.001	-0.18	[-0.29,-0.07]	0.001

Analyses of the microbiota of samples with symptomatic and asymptomatic HRV colonization and samples free of virus but with respiratory symptoms. Baseline are samples free of virus and free of symptoms.
Baseline: no virus in sample and no symptoms (n = 241); no virus in sample but with symptoms (n=77); asymptomatic HRV infection (n = 51); symptomatic HRV infection (n=69); co-infections are not included; [a]adjusted for age and season; [b]adjusted for age, season, siblings, childcare, breastfeeding, hypoallergenic nutrition, C-section, smoking in pregnancy, maternal atopy, parental education, sex
