# Supplementary material for: Interactions of Respiratory Viruses and the Nasal Microbiota during the First Year of Life in Healthy Infants
Source: mSphere. 2016 Nov 23;1(6):e00312-16. doi: 10.1128/mSphere.00312-16 (PMC5120172; doi:10.1128/mSphere.00312-16)
Supplement: Table S5 [file sph006162193st5.pdf]

Table S5: unadjusted and adjusted analysis of the association of viruses “other than HRV” with the microbiota in the same sample

	unadjusted model			adjusted model[a]			adjusted model[b]		
Outcome	IRR/ Coef	95% CI	p	IRR/ Coef	95% CI	p	IRR/ Coef	95% CI	p
PCRconc									
no symptoms	0.96	[0.76,1.22]	0.75	0.96	[0.76,1.21]	0.71	0.9	[0.71,1.14]	0.39
plus symptoms	1.15	[0.88,1.51]	0.31	1.08	[0.82,1.42]	0.59	1.07	[0.82,1.40]	0.61
SDI									
no symptoms	0.08	[-0.03,0.20]	0.137	0.09	[-0.02,0.20]	0.094	0.1	[-0.02,0.21]	0.093
plus symptoms	0.05	[-0.09,0.19]	0.477	0.07	[-0.06,0.21]	0.293	0.05	[-0.08,0.19]	0.438
Corynebacteriaceae									
no symptoms	0.95	[0.66,1.37]	0.774	1.03	[0.71,1.48]	0.893	1.15	[0.80,1.65]	0.466
plus symptoms	0.74	[0.47,1.19]	0.215	0.92	[0.57,1.47]	0.715	0.88	[0.55,1.40]	0.589
Moraxellaceae									
no symptoms	1.16	[0.86,1.56]	0.344	1.12	[0.83,1.52]	0.468	1.23	[0.91,1.65]	0.174
plus symptoms	1.07	[0.75,1.53]	0.697	1	[0.70,1.44]	0.989	0.96	[0.68,1.36]	0.807
Pasteurellaceae									
no symptoms	0.94	[0.62,1.42]	0.757	0.96	[0.63,1.46]	0.852	0.96	[0.63,1.47]	0.861
plus symptoms	1.01	[0.62,1.63]	0.973	0.89	[0.54,1.46]	0.649	0.97	[0.59,1.60]	0.903
Staphylococcaceae									
no symptoms	0.68	[0.46,1.02]	0.064	0.72	[0.48,1.08]	0.116	0.74	[0.49,1.11]	0.147

plus symptoms	0.61	[0.36,1.03]	0.062	0.74	[0.43,1.26]	0.264	0.76	[0.44,1.31]	0.318
Streptococcaceae									
no symptoms	0.84	[0.62,1.13]	0.238	0.88	[0.65,1.19]	0.419	0.93	[0.69,1.26]	0.635
plus symptoms	1.01	[0.72,1.42]	0.943	1.11	[0.79,1.57]	0.547	1.12	[0.79,1.57]	0.529
Carnobacteriaceae									
no symptoms	0.9	[0.60,1.35]	0.595	0.86	[0.57,1.30]	0.466	0.89	[0.59,1.36]	0.596
plus symptoms	0.65	[0.37,1.17]	0.151	0.67	[0.37,1.20]	0.18	0.67	[0.37,1.19]	0.172
Others									
no symptoms	1.25	[0.97,1.61]	0.089	1.19	[0.92,1.54]	0.177	1.35	[1.07,1.71]	0.013
plus symptoms	0.98	[0.71,1.36]	0.918	0.94	[0.68,1.31]	0.724	1.11	[0.81,1.53]	0.512

Analyses of the microbiota of samples with symptomatic and asymptomatic viral colonization, HRV excluded. Baseline are samples free of virus.
Baseline: no virus in sample (n = 318); asymptomatic virus infection “other than HRV” (n = 56); symptomatic virus infection “other than HRV” (n=38); aadjusted for age and season; badjusted for age, season, siblings, childcare, breastfeeding, hypoallergenic nutrition,
C-section, smoking in pregnancy, maternal atopy, parental education
