# Supplementary material for: Interactions of Respiratory Viruses and the Nasal Microbiota during the First Year of Life in Healthy Infants
Source: mSphere. 2016 Nov 23;1(6):e00312-16. doi: 10.1128/mSphere.00312-16 (PMC5120172; doi:10.1128/mSphere.00312-16)
Supplement: Table S6 [file sph006162193st6.pdf]

Table S6: unadjusted and adjusted analysis of the association of the microbiota within three weeks before symptomatic and asymptomatic HRV infection

	unadjusted model			adjusted model[a]			adjusted model[b]		
Outcome	IRR/ Coef	95% CI	p	IRR/ Coef	95% CI	p	IRR/ Coef	95% CI	p
PCRconc									
no symptoms	0.99	[0.98,1.01]	0.372	0.99	[0.98,1.01]	0.369	0.98	[0.97,1.00]	0.12
plus symptoms	1	[0.99,1.02]	0.441	1	[0.99,1.02]	0.452	1	[0.99,1.01]	0.918
SDI									
no symptoms	1.14	[0.48,2.70]	0.763	1.09	[0.44,2.72]	0.85	1.52	[0.55,4.23]	0.421
plus symptoms	0.6	[0.27,1.31]	0.199	0.47	[0.20,1.07]	0.072	0.64	[0.26,1.62]	0.347
Corynebacteriaceae									
no symptoms	1	[0.97,1.03]	0.902	0.99	[0.96,1.02]	0.626	1	[0.97,1.03]	0.807
plus symptoms	0.99	[0.97,1.02]	0.621	1	[0.97,1.03]	0.853	1	[0.97,1.04]	0.771
Moraxellaceae									
no symptoms	1	[0.99,1.01]	0.872	1	[0.99,1.01]	0.764	1	[0.98,1.01]	0.685
plus symptoms	1	[0.99,1.01]	0.46	1	[0.99,1.01]	0.37	1	[0.99,1.01]	0.883
Pasteurellaceae									
no symptoms	1	[0.97,1.03]	0.91	1	[0.97,1.03]	0.978	1	[0.96,1.04]	0.916
plus symptoms	0.97	[0.92,1.03]	0.32	0.97	[0.91,1.03]	0.312	0.97	[0.91,1.03]	0.351
Staphylococcaceae									
no symptoms	1.01	[1.00,1.03]	0.169	1.01	[0.99,1.02]	0.346	1.01	[1.00,1.03]	0.108
plus symptoms	1	[0.98,1.02]	0.848	1	[0.98,1.02]	0.798	1	[0.98,1.02]	0.977

Streptococcaceae									
no symptoms	1	[0.98,1.01]	0.557	0.99	[0.98,1.01]	0.511	1	[0.98,1.02]	0.767
plus symptoms	1.01	[1.00,1.02]	0.22	1.01	[1.00,1.02]	0.166	1.01	[1.00,1.02]	0.179
Carnobacteriaceae									
no symptoms	0.95	[0.83,1.09]	0.457	0.92	[0.79,1.08]	0.322	0.92	[0.77,1.10]	0.347
plus symptoms	0.93	[0.81,1.07]	0.342	0.92	[0.78,1.08]	0.287	0.96	[0.82,1.11]	0.541
Others									
no symptoms	1	[0.99,1.01]	0.97	1	[0.99,1.01]	0.984	1	[0.99,1.01]	0.953
plus symptoms	0.99	[0.98,1.01]	0.324	0.99	[0.98,1.00]	0.17	0.99	[0.98,1.01]	0.303

Analysis of the association of the microbiota within three weeks before symptomatic and asymptomatic HRV colonization, included are only samples free of virus before HRV colonization. Baseline are samples free of virus and no viral colonization three weeks after analysis.

Outcome parameter: Baseline: no virus in sample (n = 188); asymptomatic RV infection (n=27); symptomatic HRV infection (n=35); co-infections are not included.

Exposure: Microbiota within three weeks before, if there is no virus detected; [a]adjusted for age and season; [b]adjusted for age, season,
siblings, childcare, breastfeeding, hypoallergenic nutrition, C-section, smoking in pregnancy, maternal atopy, parental education, sex
